# Identification of Key Aroma Compounds Associated with Olfactory Perception and Pleasantness in Processed Ginseng Products: Insights from GC-MS, Flavoromics, and Computational Modeling

**DOI:** 10.3390/foods15081337

**Published:** 2026-04-12

**Authors:** Yongxu Yuan, Minjing Zhang, Yu Dong, Ming Li, Shichun Pei, Yu Xu, Yanyan Cui

**Affiliations:** 1 Jilin Province Changbai Mountain Edible Plant Resources Development Engineering Centre, Antibody Development Jilin Province University Enterprise Joint Technology Innovation Labs, Tonghua Normal University, Tonghua 134002, China; yuanyongxu@thnu.edu.cn (Y.Y.); 18626593645@163.com (M.Z.); lidaoen@163.com (M.L.); peishichun@thnu.edu.cn (S.P.); 2 Jilin Ginseng Research Institute, Tonghua 134002, China; dong9803@163.com; 3 College of Food and Health, Zhejiang A&F University, Hangzhou 311300, China

**Keywords:** SPME-GC-MS, network pharmacology, molecular docking, molecular dynamics simulation

## Abstract

The unique aroma of ginseng is linked to its recognized mood-enhancing properties. However, the specific aromatic compounds responsible for this effect, as well as the underlying mechanisms across different processed ginseng products, remain unclear. Here, the characteristic pleasant aroma compounds and their potential associations in five preparations—fresh ginseng, white ginseng, Dali ginseng, red ginseng, and black ginseng—were analyzed using flavoromics, bioinformatics, and computational modeling. The aroma evolved from “green” to “roasted-medicinal” notes, with pleasantness peaking in red ginseng, highlighting moderate processing as a key factor. Eight key pleasant aroma compounds were identified (including octanal and β-selinene), which were found to be potentially associated with olfactory- and emotion-related pathways involving IGF1 and OR6A2. Molecular interaction analysis revealed that these compounds may synergistically modulate pleasantness through hydrogen bonding and hydrophobic interactions. Furthermore, aroma harmony proved more decisive than aroma intensity in determining consumer preference, suggesting correlational evidence linking molecular interactions to sensory perception. Dynamic simulations further demonstrated stable interactions between β-selinene, octanal, and IGF1/OR6A2. This research offers new insights into the mood-modulating properties of ginseng aroma and may inform future studies exploring the development of specialized ginseng products for emotional well-being applications.

## 1. Introduction

Ginseng is a renowned traditional Chinese medicinal herb, whose diverse processing techniques yield distinct product categories including fresh ginseng, white ginseng, Dali ginseng, red ginseng, and black ginseng [1,2]. These processing methods significantly alter the chemical composition and sensory characteristics of ginseng, particularly manifesting marked differences in volatile organic compound (VOC) profiles [3]. Characteristic aromas serve not only as crucial indicators for quality assessment but, as recent studies suggest, may also participate in emotional regulation via the olfactory system, particularly in inducing pleasurable sensations [4]. However, aroma perception involves multiple olfactory receptors and complex neural networks, and the molecular mechanisms by which these compounds generate pleasurable sensations remain incompletely elucidated.

Elucidating the chemical basis of ginseng’s aroma is a crucial prerequisite for mechanistic studies. Gas chromatography-mass spectrometry (GC-MS) serves as a key tool for analyzing such complex volatile profiles [5,6]. Research indicates that fresh ginseng exhibits a fresh grassy and earthy aroma, primarily derived from aldehydes, alcohols, and certain terpenes. White ginseng exhibits a refined, subtly sweet aroma characterized by ginseng-specific terpenes and certain esters. The distinctive sweetness and roasted aroma of red ginseng is associated with its rich content of maltol and cyclic terpenes, whereas the caramelized and medicinal notes of black ginseng are closely linked to the accumulation of high levels of furans, pyrroles, and aromatic compounds [7]. These aromatic characteristics are intrinsically linked to chemical reactions occurring during processing, such as the Maillard reaction, Strecker degradation, and terpenoid transformations [8], providing robust chemical evidence for identifying core VOCs in various ginseng products.

The physiological mechanisms by which aromas influence mood via olfactory pathways have been extensively explored. For aromatic molecules to modulate emotions, they must first be effectively perceived by the olfactory system, a process initiated by their specific binding to nasal olfactory receptors [9]. Molecular docking techniques have become a standard method for predicting the interaction potential between VOCs and olfactory receptors [9,10]. For instance, in osmanthus fragrance research, integrating GC-MS analysis with molecular docking enabled researchers to successfully predict binding patterns of key aroma components with olfactory receptors, establishing a biomimetic predictive model linking chemical composition to olfactory perception [11]. Similarly, docking analysis of blue lotus fragrance components revealed the receptor-binding mechanism underlying its distinctive aroma [12]. It has been suggested that certain aroma compounds may activate olfactory receptors, which in turn could influence the release of neurotransmitters such as dopamine, serotonin, and endorphins through a cascade of signaling events within the limbic system. However, it is important to note that this process involves multiple intermediate steps, indirect pathways, and modulatory factors, and direct causal links between specific VOCs and neurotransmitter release have not been firmly established [12]. Given this complexity, investigating the potential relationships between characteristic aroma compounds in ginseng products and their olfactory perception represents a necessary first step toward understanding their possible mood-related functions.

To this end, this study established an integrated framework combining flavoromics and computational modeling with an exploratory scope to investigate potential associations between the characteristic aromas of different ginseng products and olfactory perception as well as perceived pleasantness. Specifically, GC-MS was employed to identify volatile organic compounds (VOCs) in ginseng products subjected to various processing methods, and chemometric approaches such as orthogonal partial least squares discriminant analysis (OPLS-DA) were applied to pinpoint their distinctive aroma compounds. Through volatile profiling, flavor wheel construction, quantitative descriptive analysis (QDA), and principal component analysis (PCA), the evolutionary patterns of aroma characteristics were delineated. Subsequently, network pharmacology, molecular docking, and molecular dynamics simulations were integrated to explore the interactions between these aroma compounds and human olfactory receptors, as well as their downstream pleasantness-related signaling pathways. This integrated approach provides preliminary insights into the potential principles underlying aroma-associated emotional responses and offers candidate molecular targets for future research exploring the development of functional ginseng products.

## 2. Materials and Methods

### 2.1. Ginseng Samples

Fresh ginseng samples were procured from the Jianan Qinghe Aoyang Wild Ginseng International Trading Centre in China. These comprised 5-year-old cultivated ginseng samples from the same batch, free from pests and diseases. After washing, the surface moisture was blown dry, and the samples were designated as FG. The fresh ginseng was then naturally air-dried until its moisture content fell below 12%, yielding white ginseng samples designated as WG. Fresh ginseng was blanched in 95°C hot water for approximately 10 min until the main root softened slightly, then immediately immersed in cold water for cooling. After air-drying and oven-drying, the vigorously Dali ginseng sample was produced, designated as DG. Fresh ginseng was steamed under steam conditions, then dried in a 70°C drying chamber until the moisture content fell below 12%, yielding the red ginseng sample, designated as RG. Fresh ginseng underwent nine cycles of repeated steaming and sun-drying to produce black ginseng samples, denoted as BG. These were analyzed in the subsequent step.

### 2.2. Analysis of Volatile Compounds by SPME-GC-MS

Analysis of volatile components was performed using solid-phase microextraction (SPME) coupled with an ISQ1300 gas chromatography-mass spectrometry (GC-MS) system (Thermo Fisher Scientific, Waltham, MA, USA). Specifically, 2.0 g of precisely weighed processed ginseng product was placed in a 20 mL headspace vial. (Nantong Tianxing Experimental Technology Co., Ltd., Nantong, China). All samples were equilibrated at 75 °C on a solid-phase microextraction stirring heating platform (Biotage, Uppsala, Sweden) for 30 min. Desorption was performed at 85 °C using a 65 μm divinylbenzene/polydimethylsiloxane (DVB/PDMS) extraction fiber (Supelco, Bellefonte, PA, USA) in the headspace for 30 min. Subsequently, the extraction fiber was desorbed at 260 °C for 3 min at the GC-MS inlet (Thermo Fisher Scientific, Waltham, MA, USA), and samples were injected in splitless mode. Each sample was analyzed in triplicate.

Gas chromatography separation was performed on a TR-5MS quartz capillary column (30 m × 0.25 mm × 0.25 μm) (Thermo Fisher Scientific, Waltham, MA, USA) using high-purity helium (99.999%) as the carrier gas at a flow rate of 1.0 mL/min. The column oven temperature program was as follows: initial temperature 60 °C, held for 3 min; ramped at 2 °C/min to 120 °C, held for 1 min; further ramped at 10 °C/min to 250 °C, held for 1 min; and finally ramped at 20 °C/min to 280 °C.

Mass spectrometry analysis employed electron impact (EI) mode with an ionization energy of 70 eV. Both the ion source temperature and transfer line temperature were set to 230 °C. Mass spectral data were acquired in full scan mode with a mass range of *m*/*z* 20–600 aum.

Identification of volatile compounds was based on retention time (RT) and mass spectra matching against the NIST standard library, with confirmation from relevant literature. Relative content (%) of each component was calculated using peak area normalization.

The sensory thresholds for each volatile component were obtained from relevant literature [13,14,15]. Using à-Pinene as the standard reference, its relative odor activity value (ROAV) was set to 100. The ROAV for each of the remaining compounds was then calculated according to the following formula [16]:

(1) $${\mathrm{ROAV}}_{i}\;=\;100\;\times \;\frac{C_{i}}{T_{i}}\;\times \;\frac{T_{\max}}{C_{\max}}$$

where C_i_ denotes the relative content of the target compound in the sample (%); T_i_ represents the odor threshold of the target compound (μg/kg); C_max_ denotes the relative content of the most abundant volatile compound in the sample (%); T_max_ represents the odor threshold of the most abundant volatile compound (μg/kg); and the constant 100 is the assigned ROAV of the reference compound (à-Pinene).

### 2.3. QDA Experimental Procedure

QDA was conducted by 10 trained assessors (5 males, 5 females; aged 20–35) to evaluate five ginseng products. All assessors were researchers of Chinese herbal medicines with no known drug allergies and over 120 h of experience in ginseng sensory evaluation, followed by 48 h of targeted training on ginseng aroma to ensure consistency. The sensory descriptors included olfactory attributes (green, toasty, sweet, woody, and medicinal) and pleasantness attributes (pleasantness, harmony, intensity, purity, and overall liking). Each attribute was scored on a 0–5 intensity scale (0 being imperceptible and 5 being extremely strong). To ensure the reliability and reproducibility of the results, a double-blind condition was implemented with samples coded using random three-digit numbers. The presentation order was independently randomized for each assessor using a Latin square design to balance carry-over and order effects. Each sample was evaluated in triplicate. One-way ANOVA was performed to confirm panel reproducibility across the five ginseng products. All assessors provided written informed consent, and the study adhered to ethical principles for sensory evaluation research, including respondent rights and confidentiality [17].

### 2.4. Screening of Key Volatile Compound-Related Targets and Functional Enrichment Analysis

Based on the established OPLS-DA model and ROAV metric, key aroma compounds meeting both VIP ≥ 1 and ROAV ≥ 1 across five ginseng products were identified. These compounds are presumed to play a significant role in shaping the overall olfactory characteristics and pleasantness of the products. Their SMILES structures were obtained via Open Babel GUI, and potential target predictions and collection were performed using SwissTargetPrediction, CTD, TCMSP 3.0, and PharmMapper databases. To ensure prediction confidence, targets with a probability score ≥ 0.7 (SwissTargetPrediction) or supported by at least two databases were retained as ginseng aroma targets. A two-step, hypothesis-driven search was performed in the UniProt database (https://www.uniprot.org): first using “olfactory” (olfactory-related targets), then using “dopamine,” “opioid,” and “serotonin” based on established pleasure neurocircuits (pleasure-related targets). Core targets were defined as two sets of intersections: ginseng aroma targets intersecting with olfactory-related targets, and ginseng aroma targets intersecting with pleasure-related targets. We acknowledge that this keyword-based approach is hypothesis-driven rather than unbiased. This is explicitly justified by the study’s focus on known pleasure neurocircuits, and its limitations are addressed later in this paper.

Next, the selected core targets were imported into the STRING platform to construct a protein interaction network. Cytoscape software (v3.10.3) was used to visualize the “active ingredient-target” relationships. The degree centrality of network nodes was calculated via the Network Analyzer plugin to assess their topological importance. Gene Ontology (GO) functional enrichment analysis and KEGG pathway enrichment analysis were performed using the “Wei Sheng Xin” online platform, with results sorted by *p*-value. The GO analysis categorized gene functions into biological processes, cellular components, and molecular functions; the KEGG analysis identified key pathways involved in the generation and regulation of pleasurable sensations. Results were presented as the top 10 most significant GO functional terms and 20 KEGG pathways.

### 2.5. Molecular Docking

The key aroma-active molecules (VIP ≥ 1 and ROAV ≥ 1) screened from ginseng products were used as ligands, with their three-dimensional structures (.mol2 format) sourced from the TCMSP 3.0 database. The ligand structure files were converted to PDB format using Open Babel GUI software (Version 3.3.1). The crystal structures of the receptor proteins (OR6A2, UniProt ID: O95222; IGF1, UniProt ID: P05019) were downloaded from the UniProt database. Receptors and ligands were preprocessed using AutoDockTools-1.5.7 software. Specific steps included: removing water molecules from the protein structure, adding hydrogen atoms, and converting the processed structure to PDBQT format. Molecular docking was performed in AutoDock Vina. During docking, the ligand molecule was set as flexible (all rotatable bonds allowed free rotation), while the receptor structure was set as rigid. A grid box was defined based on the receptor active site to cover the binding site. For OR6A2, grid boxes were centered with x ranging from −14.343 to −1.128, y = −0.401, and z ranging from 1.331 to 2.756, with a spacing of approximately 0.9–1.0 Å. For IGF1, grid boxes were centered with x = −5.727 or 4.182, y ranging from −5.753 to 6.753, and z ranging from 10.971 to 14.702, with a spacing of approximately 0.9–1.0 Å. Detailed parameters for each ligand–receptor pair are provided in Supplementary Table S1. To ensure reliability, a self-consistency test was performed by comparing binding poses across three independent runs, and the protocol was considered reliable when the pairwise root-mean-square deviation (RMSD) was less than 2.0 Å. Notably, the octanal-OR6A2 pair included in our docking analysis has been independently validated as a natural ligand–receptor pair in a recent structural study [18], supporting the biological relevance of our docking results and serving as an indirect positive control. The docking parameter “num_modes” was set to 50 to retain the 50 docking conformations with the lowest binding free energies for subsequent analysis.

### 2.6. Molecular Dynamics Simulation

Building upon the prior molecular docking findings, four representative ligand–receptor complex systems (Octanal-OR6A2, Octanal-IGF1, β-Selinene-OR6A2, and β-Selinene-IGF1) were selected for all-atom molecular dynamics (MD) simulations using YASARA v10.3.16 software. This investigation aimed to study their dynamic behavior and binding stability under near-physiological conditions. At the start of simulations, each complex underwent structural optimization and energy minimization. Subsequently, the systems were confined within cubic boxes with periodic boundary conditions, supplemented with TIP3P water molecules and 150 mM NaCl to simulate physiological ionic strength and maintain electroneutrality. Simulations proceeded under NPT conditions, with temperature and pressure controlled at 298 K and 1 bar, respectively, using the Berendsen constant temperature and pressure method. Atomic interactions were described using the AMBER force field, with long-range electrostatic forces handled via the Particle Mesh Ewald (PME) method and van der Waals interactions calculated using the truncated radius method. Each system underwent a 100 ns simulation with an integration step size of 2 fs, and trajectory coordinates were saved every 100 ps. The obtained trajectories will be utilized for subsequent analyses, primarily including: assessing the overall conformational stability of the complex based on RMSD; analyzing the local flexibility of key residues at the binding interface using root-mean-square fluctuation (RMSF); and estimating the ligand–receptor binding free energy via the MM-PBSA method. Results were visualized using Origin 2021.

### 2.7. Data Statistical Analysis

Calculations were performed using mean ± standard deviation. PCA and OPLS-DA analysis were conducted using SIMCA 14.1 software. The weighted binding energy was calculated as the product of the relative content of eight key aroma compounds and the absolute binding energy with OR6A2 and IGF1 receptors. A correlation network diagram was then generated using an online tool available at www.chiplot.online. Data visualization was completed using Origin 2021, GraphPad Prism 8, and Adobe Photoshop 12 software. Statistical significance was set at *p* < 0.05. For correlation analyses, significance levels of *p* < 0.05 and *p* < 0.01 were used to indicate moderate and strong correlations, respectively.

## 3. Results

### 3.1. Analysis of Volatile Compounds in Ginseng Products

**Figure 1:**
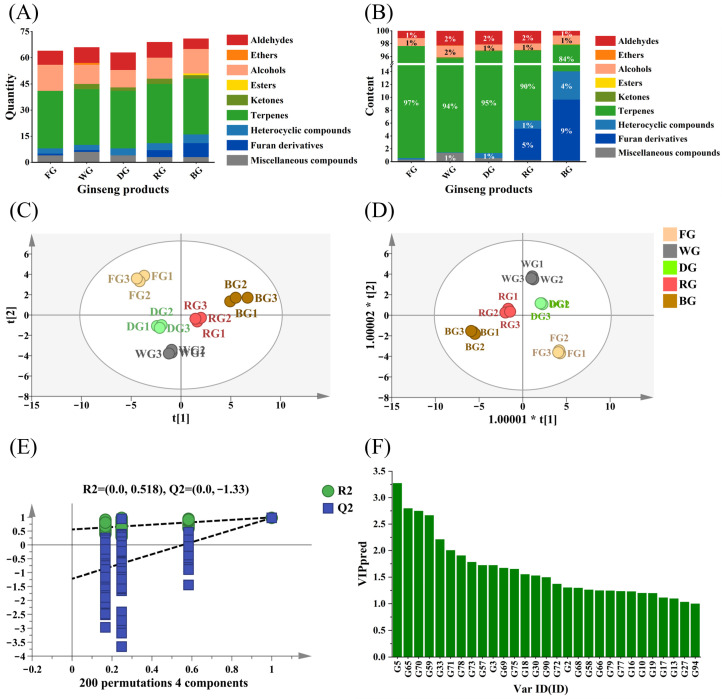
Volatile compound profiles of five ginseng products. (**A**) Stacked plot of different volatile components. (**B**) Stacked plot of percentages different volatile components. (**C**) PCA plot. (**D**) OPLS-DA model. (**E**) Hypothesis testing for the OPLS-DA model. (**F**) VIP values.

The volatile components of ginseng products are significantly influenced by processing techniques, with their aromatic characteristics resulting from the combined effects of multiple chemical reactions and biotransformation. This study employed SPME-GC-MS for systematic analysis of five ginseng products, identifying 105 volatile compounds across nine major categories: terpenes, alcohols, aldehydes, furans, ketones, esters, ethers, heterocyclic compounds, and others (Figure 1A and Supplementary Table S2). FG contained 64 aroma compounds. After processing, WG and DG retained similar levels with 66 and 63 aroma compounds, respectively. However, RG and BG, subjected to repeated high-temperature steaming and sun-drying, exhibited significantly increased aroma compound diversity at 69 and 71 compounds. This indicates that repeated high-temperature steaming and sun-drying effectively enhances the diversity of ginseng aroma compounds. This result is consistent with previous findings [3]. Among nine major volatile component classes (Figure 1B), terpenoids constituted the highest proportion (83.70–97.06%) in all five ginseng samples, followed by alcohols (0.93–1.79%) and aldehydes (0.72–2.28%). Furan and heterocyclic compounds exhibited higher proportions only in RG and BG, at 4.85% and 9.50% versus 1.30% and 4.40%, respectively.

Terpenes represented the most abundant chemical class among all tested samples, demonstrating that they constitute the primary contributors to the characteristic aroma profile of ginseng. FG retained the highest terpene content (97.06%), which decreased to varying degrees after different processing methods. BG, subjected to repeated steaming and drying, showed a terpene content reduction to 83.70%. This change primarily resulted from the following mechanisms: first, terpenoid compounds readily undergo bond cleavage and structural rearrangement at high temperatures, leading to decomposition or participation in the Maillard reaction; second, direct loss through volatilization during processing; and third, the hydrothermal environment promoting the leaching or hydrolysis of certain components. These patterns align with earlier work [19], collectively revealing the multi-pathway depletion of terpenoids during thermal processing. Concurrently, terpenoids constitute the primary volatile components of aromatic plants, and their rich structural diversity provides the essential chemical foundation for species-specific aromatic characteristics [20]. In ginseng products, the diversity of terpenoid components manifests as the presence of multiple isomers and their derivatives, such as β-selinene and γ-selinene. This structural diversity likely originates from complex biosynthetic networks, including the synergistic interaction between the mevalonate pathway (MVA) and the methyl erythritol phosphate pathway (MEP) [21]. These metabolic pathways jointly regulate the synthesis and accumulation of monoterpenes and sesquiterpenes in ginseng through multiple key enzymatic reactions, thereby shaping its distinctive volatile aroma profile.

Furan compounds, as characteristic thermal processing markers, are aromatic heterocyclic compounds whose content significantly increased in RG and BG (4.85% and 9.50%, respectively), exhibiting an upward trend with increasing steaming and sun-drying cycles. Their formation positively correlates with the Maillard reaction intensity, primarily associated with sugar isomerization and high-temperature degradation [22,23]. The major furan compounds detected—furfural, furfuraldehyde, and 5-methylfurfural—are typical thermal processing markers widely present in various food systems [7]. Their structural variations primarily involve substituent type and position: furfural bears a hydroxyl group on the furan ring side chain without ring methyl groups; 5-methylfurfural contains a methyl group at the C5 position; while furfuraldehyde lacks methyl substituents entirely. These structural variations confer distinct reactivity and flavor profiles, collectively imparting the characteristic roasted and caramelized sensory attributes of RG and BG [24]. Among these, 5-methylfurfural, with its pronounced caramel aroma, is often regarded as a key contributor to the roasted flavor—demonstrating how specific metabolites significantly shape the sensory properties of the final product.

Analysis of alcoholic compounds revealed that (Z)-3-hexen-1-ol, 1-hexanol, 1-octanol, and 2-decen-1-ol are unique to FG. In contrast, the sesquiterpenol content in the other four ginseng products was higher than that in FG. This differential distribution primarily stems from two factors: first, the abundant sesquiterpene precursors inherent in ginseng provide ample substrate for alcohol conversion; second, distinct processing methods directly regulate the efficiency and pathways of hydroxylation reactions, thereby reshaping the final alcohol composition [25,26]. Aldehyde compositions varied across ginseng products, yet thermal processing universally induced specific accumulation of unsaturated aldehydes (E)-2-nonenal and (E)-2-decenal. This trend was particularly pronounced in RG and BG samples, with BG additionally exhibiting (E)-2-octenal formation. This process thermodynamically favors the formation of the stable trans configuration and is synergistically driven by lipid oxidative degradation and the Maillard reaction.

The PCA score plot of volatile components in different ginseng products (Figure 1C) shows distinct sample distributions: FG occupies the second quadrant alone; DG and WG cluster together in the third quadrant, suggesting chemical composition similarities; while RG and BG predominantly occupy the first and fourth quadrants, respectively. This result clearly reflects the overall differences in volatile component profiles among ginseng products resulting from distinct processing methods. Further OPLS-DA analysis (Figure 1D) revealed: R^2^X = 0.977, R^2^Y = 0.994, and Q^2^ = 0.967. All values approached one, indicating the model possesses high variance-explaining capability and excellent predictive performance. Simultaneously, the scatter plot of the five sample groups showed good clustering, with minimal intra-group variation and complete separation between groups. Validation via 200 permutations (Figure 1E) confirmed no overfitting (R^2^ = 0.518, Q^2^ = −1.33), indicating structural robustness suitable for subsequent differential volatile compound screening. A total of 29 differentially volatile components with VIP values ≥ 1 were identified (Figure 1F), serving as key volatiles distinguishing the aroma profiles of different ginseng products. Concurrently, ROAV analysis (Table 1) further identified 15 key flavor compounds with ROAV ≥ 1, including hexanal (fruity, herbal, and woody notes). Six additional compounds with ROAV values between 0.1 and 1 contributed minimally to overall flavor, primarily acting as modifiers. Collectively, these substances determine the unique sensory characteristics of each ginseng product.

**Figure 2:**
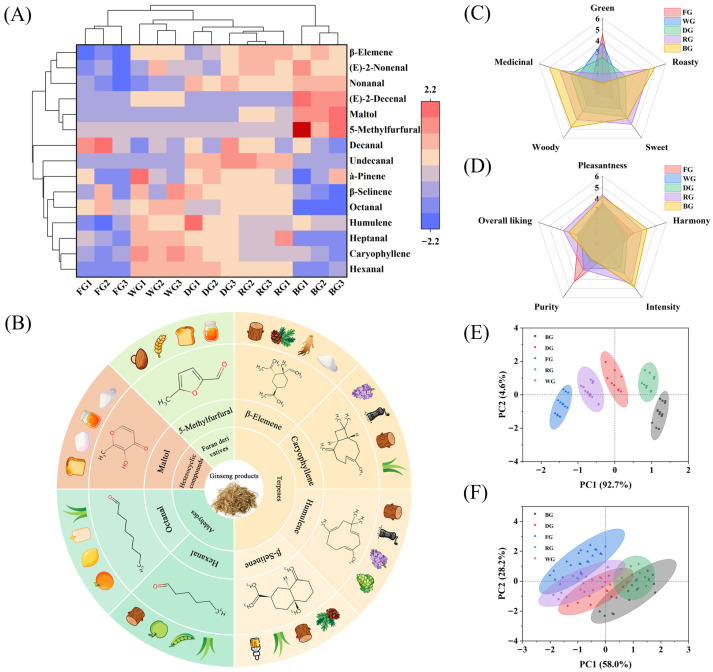
Comprehensive analysis of flavor profiles and sensory evaluation of five ginseng products. (**A**) Heatmap analysis. (**B**) Flavor wheel construction. (**C**) Radar chart of QDA olfactory sensory evaluation. (**D**) Radar chart of QDA pleasantness sensory evaluation. (**E**) PCA of olfactory sensory evaluation. (**F**) PCA of pleasantness sensory evaluation.

A heatmap analysis of characteristic aroma components in ginseng products was conducted based on the relative abundance of key aroma compounds with ROAV values exceeding 0.1, visually revealing differences in aroma profiles among various ginseng products. Results (Figure 2A) show that this clustering structure reflects the significant impact of different processing methods on the overall aroma profiles of ginseng products; the color gradient from blue to red indicates increasing concentrations of volatile compounds. BG clustered separately due to its rich content of key aroma components such as (E)-2-decenal, (E)-2-nonenal, maltol, 5-methylfurfural, and nonanal, exhibiting a complex aroma profile dominated by caramel and toasted bread notes, blended with woody, earthy, floral, and fatty aromas. The other four categories are grouped together due to similar aromatic compositions. The relatively high relative content of decanal, β-selinene, and octanal in FG collectively imparts its characteristic aroma profile, dominated by citrus notes and a fatty quality, accompanied by herbal and medicinal undertones. The predominant octanal, caryophyllene, humulene, heptanal, and hexanal in WG impart a fresh, fruity, and herbal aroma with underlying fatty and woody undertones. The relatively high levels of β-elemene, (E)-2-nonenal, and undecanal in RG provide a foundation of herbal and fresh fruit notes, integrated with floral, soapy, and fatty undertones. This classification pattern clearly reflects the decisive role of processing techniques in aroma evolution.

Through intersection analysis (VIP ≥ 1 and ROAV ≥ 1), eight potential bioactive volatile compounds were identified from five ginseng products, including hexanal, octanal, maltol, β-elemene, humulene, caryophyllene, β-selinene, and 5-methylfurfural. A characteristic aroma flavor wheel was constructed (Figure 2B) to visually map the distribution of these compounds across products, thereby establishing a direct link between molecular composition and sensory characteristics and effectively revealing the distinct aromatic profiles of each product.

### 3.2. QDA Analysis

Five sensory descriptors were used to evaluate the olfactory and pleasantness attributes of five ginseng products. One-way ANOVA revealed significant differences among the five ginseng products for multiple sensory attributes (*p* < 0.05) in Supplementary Table S3, confirming the panel’s ability to discriminate between samples. Olfactory QDA (Figure 2C) revealed a systematic flavor evolution from “green dominance” to “roasted–medicinal dual dominance” with increasing processing intensity from FG to BG. Specifically, FG exhibited prominent green notes with faint sweet, woody, and medicinal aromas; WG showed reduced green aroma with enhanced sweet, woody, and medicinal notes, along with emerging roasted notes; DG displayed further diminished green aroma with dominant sweetness, forming a balanced profile; RG exhibited low green aroma, with roasted and sweet notes as core characteristics, accompanied by intensified medicinal and woody notes; and BG showed nearly absent green aroma with peaking roasted and medicinal notes, creating a complex aroma structure. This pattern aligned with heatmap clustering and flavor wheel distributions, indicating that quantitative changes in aroma compounds drive qualitative sensory shifts. The flavor evolution is driven by multiple chemical pathways. The loss of green notes corresponds to the volatilization of low-boiling-point aldehydes and alcohols. The emergence of roasted and sweet notes arises from the Maillard reaction, which produces pyrazines and furans. Furthermore, the intensification of medicinal and woody notes is associated with the thermal degradation of ginsenosides and lignin-based components [3]. Pleasantness QDA (Figure 2D) indicated that moderate processing (RG) enhanced intensity, harmony, and overall liking, suggesting an optimal processing level for sensory acceptance. Conversely, excessive processing (BG) imparted a unique profile but caused imbalance due to pronounced roasted and medicinal notes and reduced purity, diminishing overall liking.

PCA corroborated these patterns. On the olfactory dimension (Figure 2E), PC1 and PC2 explained 92.7% and 4.6% of the variance, respectively (cumulative 97.3%), showing distinct separation and indicating flavor compound restructuring during processing. On the pleasantness dimension (Figure 2F), PC1 and PC2 explained 58.0% and 28.2% of the variance, respectively (cumulative 86.2%), with sample overlap reflecting a gradual evolution of pleasantness perception. BG exhibited exceptionally high PC2 scores, confirming its distinctiveness in pleasantness perception, although this uniqueness did not translate into higher acceptability. Collectively, these findings provide quantitative evidence for processing-driven sensory evolution among ginseng products, supporting product positioning and consumer preference analysis.

### 3.3. Screening of Key Targets and Functional Enrichment

**Figure 3:**
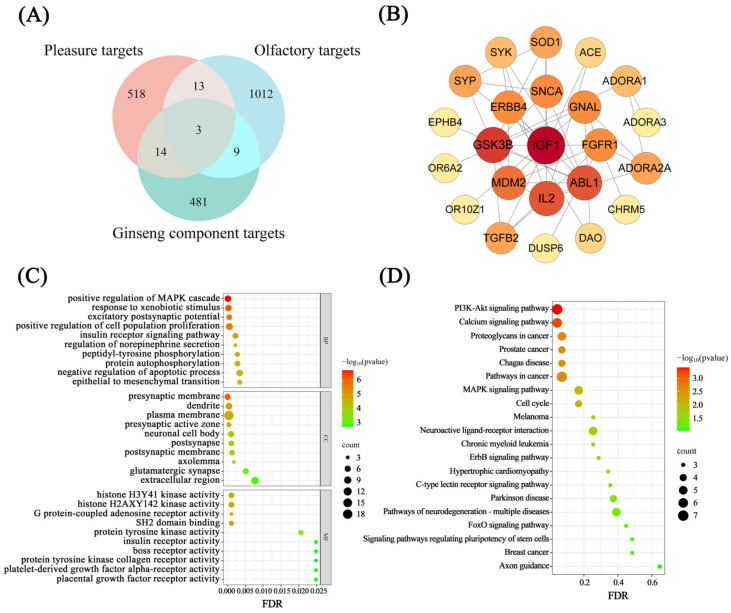
Comprehensive network pharmacology and bioinformatics analysis of bioactive compounds in ginseng products. (**A**) Venn diagram of key targets; (**B**) PPI network diagram (node color: red → yellow, red = higher degree centrality); (**C**) GO enrichment analysis of key targets; and (**D**) KEGG enrichment analysis of key targets.

To investigate the potential molecular mechanisms underlying olfactory perception and pleasantness regulation by ginseng aroma compounds, we predicted targets for eight volatile components using SwissTargetPrediction, yielding 507 potential target genes (Supplementary Table S4). Additionally, 699 pleasantness-associated and 1037 olfactory-related protein targets were retrieved from the UniProt database. Intersection analysis of these datasets (Figure 3A, Supplementary Table S3) identified 26 common targets, suggesting that ginseng volatile components may collectively act on these key targets to regulate olfactory perception and pleasantness.

A protein interaction network was constructed based on these 26 intersecting targets (Figure 3B), comprising 23 nodes and 45 interaction edges, with individual targets hidden. Node size is proportional to degree values. Key hub targets include IGF1, GSK3B, IL2, ABL1, MDM2, FGFR1, GNAL, SNCA, and ERBB4. Among these, IGF1 exhibited the highest degree centrality in the PPI network, indicating a prominent topological position. Functionally, IGF1 has been implicated in both neuroplasticity and olfactory pathways [27,28]. It supports emotional regulation by promoting neuronal survival, synapse formation, and neuroplasticity, and has been associated with affective disorders such as depression [27]. In the olfactory system, IGF1 is involved in axonal projection of olfactory sensory neurons to the olfactory bulb, facilitating olfactory map construction and signal transmission [28]. Moreover, olfactory experience can upregulate IGF1 expression in the brain, suggesting its role in integrating sensory and emotional information [29]. Concurrently, other hub targets such as FGFR1 (involved in emotional behavior regulation and olfactory system maintenance) and GNAL (involved in olfactory signal transduction and dopamine reward pathways) may exert synergistic effects in olfactory–emotional integration [30,31]. In addition, two olfactory receptors, OR6A2 and OR10Z1, appeared in the network, indicating that olfactory receptors are not isolated but integrated into a broader interaction network involving IGF1 and other signaling proteins. Notably, OR6A2 exhibits broad-spectrum ligand recognition and structural plasticity, including the ability to form reversible covalent bonds with odorants, making it a representative model for studying complex aroma components [18]. Based on these findings, the relatively high centrality of IGF1 in the network and the functional representativeness of OR6A2 in aroma perception provide a rationale for selecting these two proteins as core targets for subsequent molecular docking analyses.

GO and KEGG pathway enrichment analysis revealed the biological functions of these 26 overlapping targets. GO analysis results (Figure 3C) showed that these targets were significantly enriched in 118 biological processes, 22 cellular components, and 39 molecular functions, accounting for 65.92%, 12.29%, and 21.79% of all enriched entries, respectively. Within biological processes, the selected key targets predominantly enriched signaling pathways associated with neurotrophic factors and neuroactive signaling molecules, including positive regulation of the MAPK cascade, excitatory postsynaptic potential, and regulation of norepinephrine secretion. Key signaling pathways like MAPK promote neuronal survival, differentiation, and synaptic plasticity, playing crucial roles in learning, memory, and the formation of positive emotions [32,33]. Additionally, some targets participate in processes such as response to xenobiotic stimulus, the insulin receptor signaling pathway, and peptidyl-tyrosine phosphorylation and protein autophosphorylation. These mechanisms are widely present in intracellular signaling networks. Within the context of olfactory perception and emotional regulation, they may synergistically participate in neural function regulation by modulating the activation of downstream signals from neurotrophic factor receptors (e.g., TrkB) and G protein-coupled receptors (GPCRs) [34].

Within cellular compartment categories, key targets are primarily localized to neuron-associated structures. Among these, the presynaptic membrane, dendrite, postsynapse, postsynaptic membrane, and glutamatergic synapse are functionally central to olfactory perception and the formation of pleasurable emotions [35]; other structures predominantly serve supportive roles in maintaining basic neuronal structure and functional stability. In the molecular function category, relevant targets predominantly cluster within three major functional modules: neural signaling and regulation, cell growth and metabolic regulation, and epigenetic regulation. Specifically, significant enrichment was observed in key signaling functions such as G protein-coupled adenosine receptor activity, insulin receptor activity, protein tyrosine kinase activity, and SH2 domain binding. Additionally, enrichment was observed in functions including histone H3Y41 kinase activity, histone H2AXY142 kinase activity, boss receptor activity, and protein tyrosine kinase collagen receptor activity.

Based on KEGG pathway analysis (Figure 3D), the key targets identified in this study were significantly enriched in multiple core signaling pathways, including neuroactive ligand–receptor interactions, calcium signaling, MAPK signaling, and PI3K–Akt signaling. Notably, the neuroactive ligand–receptor interactions pathway encompasses not only olfactory receptors belonging to the GPCR family but also involves key neurotransmitter systems crucial for regulating pleasurable emotions, such as dopamine receptors, serotonin receptors, GABA receptors, and glutamate receptors. The binding of volatile components to their corresponding receptors constitutes the initial step in triggering olfactory perception, while the formation of pleasurable emotions relies on the precise regulation of these neurotransmitters within the limbic system [4]. At the signal transduction level, calcium ions (Ca^2+^) serve as a crucial second messenger. They mediate action potential generation in olfactory sensory neurons while also regulating neurotransmitter release across synapses and establishing synaptic plasticity, thereby influencing the maintenance and consolidation of pleasurable experiences [36]. Furthermore, processes such as axon guidance and the FoxO signaling pathway play supportive roles in neural development and functional maintenance, providing foundational assurance for the integrity of the olfactory-emotional pathway. Collectively, KEGG pathway and GO enrichment analyses demonstrate that the key targets identified in this study play central roles in the formation and regulation of pleasantness by participating in neural signal transduction and modulating neurotransmitter receptor activity.

### 3.4. Molecular Docking of Key Aroma Compounds with Core Targets

According to PPI network analysis, IGF1 exhibited the highest degree centrality and plays dual regulatory roles in olfactory and emotional pathways. It was thus identified as a representative hub target for investigating the integration of aroma perception and affective processing. Meanwhile, OR6A2 was selected not only for its presence in the network, but also for its well-documented broad-spectrum recognition of aroma components and structural plasticity [31,37,38]. Recent studies further indicate that OR6A2 can recognize aldehyde molecules through reversible covalent bonds, highlighting the high ligand diversity and structural adaptability of olfactory receptors [18]. This provides a theoretical basis for studying the small molecule binding characteristics of this receptor. Based on these considerations, IGF1 and OR6A2 were chosen as receptor models to investigate the binding properties of eight key aroma molecules in ginseng products.

Molecular docking results (Table 2) showed that all pairwise RMSD values were below 2.0 Å (range: 0–1.32 Å), confirming the stability and reproducibility of the docking protocol across independent runs. The results also revealed distinct amino acid binding sites within each ligand–receptor complex and uniformly negative binding energies, indicating that the binding between receptor proteins and aroma compounds occurs spontaneously and stably. Binding energy is commonly used to evaluate the strength of interactions between ligands and receptors: lower binding energy (i.e., more negative values) typically reflects stronger affinity and higher structural stability of the complex [39]. Comparing binding energies between the two receptors and each compound revealed that the binding energies for the eight key aroma-active molecules in OR6A2 were consistently lower than their corresponding values in IGF1, suggesting OR6A2 exhibits higher binding affinity for these aromatic molecules. Further analysis revealed significant variations in binding energies when the same receptor interacts with different aromatic compounds. Among these, β-Selinene-OR6A2 (binding energy: −6.93 kcal/mol) exhibited the strongest binding affinity, while Octanal-IGF1 (binding energy: −2.65 kcal/mol) demonstrated the weakest. Therefore, in the presence of multiple aroma molecules, high-affinity compounds may preferentially occupy the limited binding sites on the receptor protein. This finding also helps explain the differences in aroma characteristics and perception thresholds exhibited by various aromatic compounds [40].

**Figure 4:**
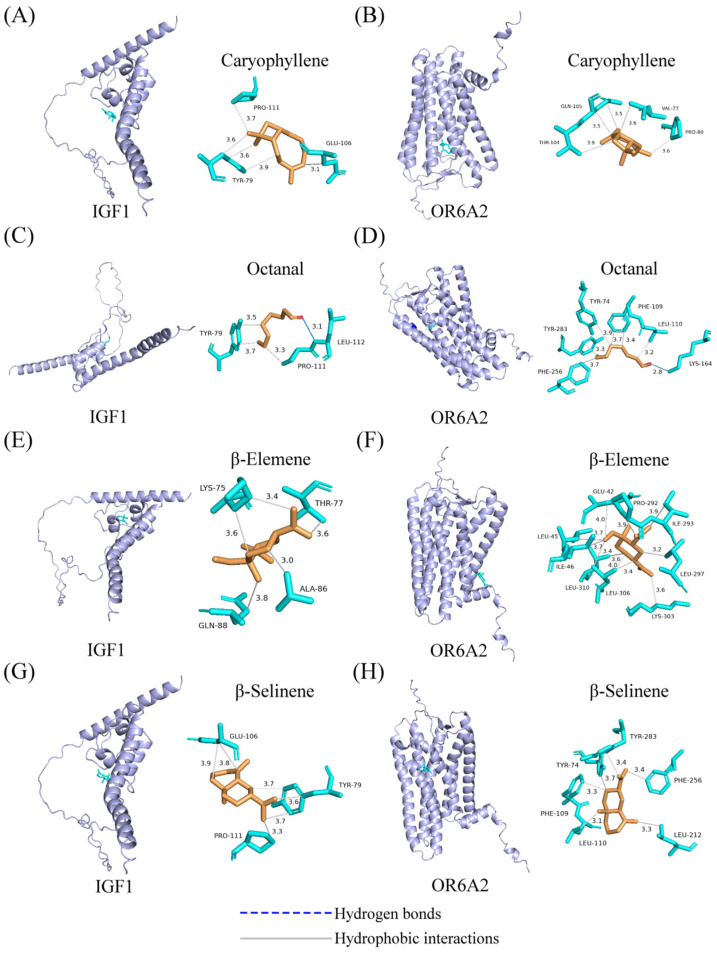
Molecular docking analysis. (**A**) Interaction diagram of the caryophyllene-IGF1 complex. (**B**) Interaction diagram of the caryophyllene-OR6A2 complex. (**C**) Interaction diagram of the octanal-IGF1 complex. (**D**) Interaction diagram of the octanal-OR6A2 complex. (**E**) Interaction diagram of the β-elemene-IGF1 complex. (**F**) Interaction diagram of the β-elemene-OR6A2 complex. (**G**) Interaction diagram of the β-selinene-IGF1 complex. (**H**) Interaction diagram of the β-selinene-OR6A2 complex.

The binding energies of eight receptor–ligand complexes, shown in Figure 4, are below −2.6 kcal/mol, indicating a strong thermodynamic driving force for their binding processes. Both hydrogen bonding and hydrophobic interactions played critical roles in the binding of aroma volatiles to the receptors. For example, octanal formed hydrogen bonds with residues LYS164 of OR6A2 and LEU112 of IGF1, respectively, while multiple hydrophobic residues in the binding pocket provided a stable hydrophobic environment that further enhanced binding stability [39]. Specifically, β-selinene formed hydrophobic interactions with residues TYR74 (3.65 Å), PHE109 (3.34 Å), LEU110 (3.08 Å), LEU212 (3.31 Å), PHE256 (3.38 Å), and TYR283 (3.44 Å) in OR6A2, and with residues TYR79 (3.58 Å, 3.73 Å, and 3.72 Å), GLU106 (3.79 Å and 3.87 Å), and PRO111 (3.31 Å) in IGF1. Caryophyllene interacted with residues VAL77 (3.64 Å), PRO80 (3.59 Å), THR104 (3.89 Å), and GLN105 (3.54 Å and 3.48 Å) in OR6A2, while in IGF1 it primarily bound to residues TYR79 (3.55 Å, 3.57 Å, and 3.89 Å), GLU106 (3.13 Å), and PRO111 (3.73 Å). β-Elemene interacted with nine hydrophobic residues in OR6A2: GLU42 (3.96 Å), LEU45 (3.74 Å), ILE46 (3.68 Å), PRO292 (3.90 Å), ILE293 (3.94 Å), LEU297 (3.22 Å), LYS303 (3.64 Å), LEU306 (3.45 Å, 3.96 Å and 3.40 Å), and LEU310 (3.58 Å); in IGF1 it bound to 4 residues: LYS75 (3.62 Å and 3.43 Å), THR77 (3.64 Å), ALA86 (2.96 Å), and GLN88 (3.77 Å). The multi-site cooperative binding pattern may confer enhanced conformational stability and persistence to the complexes, facilitating the sustained retention and recognition of aroma molecules within the olfactory system. These findings reveal the binding characteristics of active ginseng aroma components within two receptor types. Through hydrogen bonds and hydrophobic interactions, they effectively engage key targets, highlighting potential mechanisms for ginseng aroma perception and pleasure regulation. However, it should be noted that these molecular docking results only predict potential binding interactions; therefore, the proposed mechanisms remain hypothetical.

### 3.5. Correlation Analysis Between Molecular Docking Binding Energy and Sensory Evaluation

**Figure 5:**
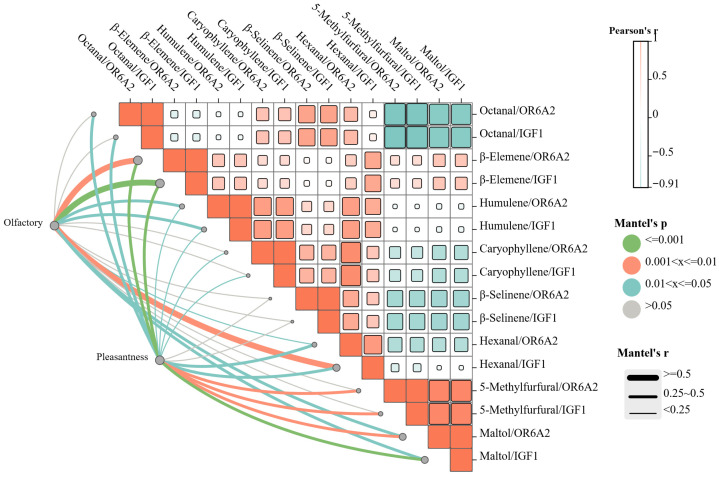
Network diagram of the correlation analysis of binding energies of aroma compounds with receptor proteins and their sensory attributes.

Based on Mantel tests and Pearson correlation analyses of weighted binding energies between eight key aroma compounds and OR6A2/IGF1, the molecular mechanisms underlying the aroma characteristics of five ginseng products were systematically elucidated. As shown in Figure 5, Mantel tests revealed significant correlations between the compound-OR6A2 binding energy matrix and the olfactory sensory attribute matrix (green, roasty, sweet, woody, and medicinal) (Mantel’s r = 0.001–0.600, *p* < 0.01), confirming that compositional differences in these compounds fundamentally determine the aroma profiles of different processed products. For example, Hexanal/OR6A2 binding energy showed a strong positive correlation with green aroma (Mantel’s r = 0.400), most pronounced in FG, while Maltol/OR6A2 binding energy positively correlated with roasted aroma (Mantel’s r = 0.600), peaking in BG.

Pearson correlation analysis further clarified the functional differentiation of the two receptors [41]: OR6A2 exhibited a significant positive correlation with aroma intensity (average Pearson’s r = 0.5)—BG achieved high intensity due to strong binding affinities with multiple compounds/OR6A2—but correlated negatively with aroma purity, with FG showing the highest purity despite lower intensity. Conversely, IGF1 showed a significant positive correlation with aroma harmony and overall liking (average Pearson’s r = 0.5), supporting its role as a receptor for pleasantness. For instance, RG exhibited high β-Elemene/IGF1 binding affinity, corresponding to high harmony and overall liking, whereas BG, despite high aroma intensity and the strongest Maltol/OR6A2 binding affinity, showed reduced overall liking due to relatively weak Maltol/IGF1 binding affinity. These findings established a correlative framework linking molecular interactions to sensory perception, with aroma harmony and receptor activation emerging as key determinants of consumer preference.

### 3.6. Molecular Dynamics Simulation of Core Ligand–Receptor Complexes

**Figure 6:**
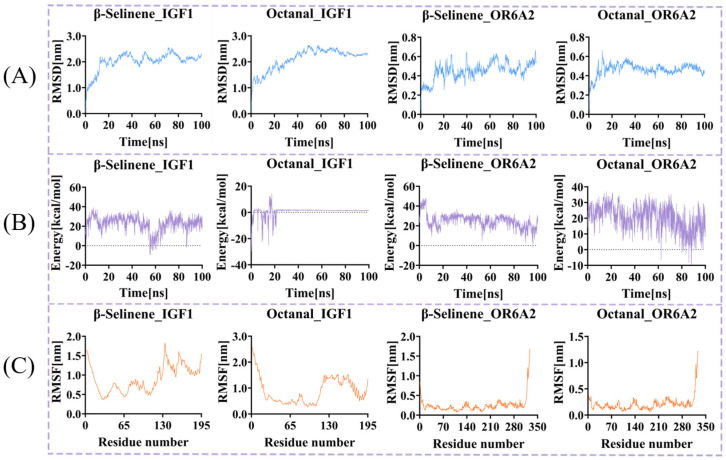
Molecular dynamics simulation analysis. (**A**) RMSD of the four complexes. (**B**) Binding energy of the four complexes. (**C**) RMSF of the four complexes.

MD simulations serve as a crucial dynamics tool for investigating conformational evolution and complex stability during protein–ligand binding processes [42]. As a commonly used metric for evaluating structural alignment discrepancies, the temporal trend of RMSD directly reflects the conformational stability of complexes during simulations [4]. Figure 6A shows that all systems exhibited an initial increase in RMSD during the early simulation phase (approximately 20–30 ns) due to solvent-induced conformational relaxation, followed by a transition into a steady-state fluctuation phase, ultimately reaching dynamic equilibrium. The average RMSDs for Octanal-OR6A2 and β-Selinene-OR6A2 stabilized around 4.641 Å and 4.501 Å, respectively, with fluctuation amplitudes progressively decreasing throughout the simulation. This indicates both ligands guide OR6A2 into forming compact and stable binding conformations. In the IGF1 complex system, the average RMSDs for the octanal and β-selinene systems were 20.968 Å and 20.782 Å, respectively, markedly higher than typical globular protein values. This discrepancy arises from three aspects: (i) IGF1 is a flexible 70-amino-acid peptide hormone with an intrinsically dynamic structure [43]; (ii) the observed RMSD primarily reflects global protein motions (domain rearrangements and terminal fluctuations) rather than local binding site stability, as supported by RMSF analysis (Figure 6C) showing pronounced fluctuations in the N/C-terminal regions; and (iii) these elevated values are consistent with IGF1’s physiological function as a conformationally adaptable signaling molecule [43]. Importantly, simulation convergence was assessed using multiple metrics beyond RMSD alone. As shown in Figure 6A, all IGF1 complexes reached dynamic equilibrium after approximately 50 ns, with RMSD trajectories fluctuating stably around a constant mean without systematic drift, indicating conformational convergence. Furthermore, the binding energies of both IGF1 complexes stabilized after 40 ns, fluctuating within a narrow range of –2 to 2 kcal/mol (Figure 6B). Together, the stable RMSD trajectories and converged binding energies provide robust evidence that the simulations were sufficiently long to capture equilibrium behavior. This indicates that octanal and β-selinene can form stable interactions with their respective target proteins, providing reliable kinetic evidence for further systematic analysis of their binding mechanisms at the atomic level.

Binding energy calculations further elucidate the dynamic optimization process of protein–ligand interactions. As shown in Figure 6B, the binding energies of Octanal-OR6A2 and β-Selinene-OR6A2 exhibited significant fluctuations during the early simulation phases (0–80 ns and 0–60 ns, respectively), ranging from −9.382 to 36.771 kcal/mol (mean 20.262 kcal/mol) and −1.843 to 47.494 kcal/mol (mean 25.016 kcal/mol), respectively, with frequent transient peaks. This reflects water molecule rearrangement at the binding interface, dynamic hydrogen bond changes, and adjustments in hydrophobic interactions [44]. As simulations progressed, the binding energies of both systems decreased overall and stabilized: Octanal-OR6A2 exhibited multiple intervals below 10 kcal/mol after 80 ns; β-Selinene-OR6A2 decreased in energy after 60 ns, reaching its minimum value (−1.843 kcal/mol) at 95.9 ns. This discrepancy likely stems from β-selinene’s larger molecular volume and stronger hydrophobicity, enabling it to more effectively fill OR6A2’s hydrophobic pocket. This facilitates more favorable van der Waals interactions and entropy contributions, consistent with studies [45,46] demonstrating how ligand physicochemical properties influence binding affinity. During the final 20 ns, trajectories of both systems converged, exhibiting significantly reduced fluctuations and stabilizing at lower energy states, indicating energetically stable binding configurations for both ligands and OR6A2. In the IGF1 complex system, the binding energy dynamics of β-Selinene-IGF1 followed this trend. In contrast, the Octanal-IGF1 binding energy evolution exhibited distinct phases: initial (0–2 ns) violent oscillations, rapidly decreasing from −15.861 kcal/mol to −29.496 kcal/mol before rebounding to positive values, indicating significant conformational rearrangement and interface reorganization after initial binding. Between 2–40 ns, fluctuations were accompanied by multiple transient negative values (e.g., −14.372 kcal/mol and −22.446 kcal/mol), indicating local conformational searches and competitive interactions. A sustained positive phase (peaking at 14.018 kcal/mol) emerged between 15–17 ns, potentially linked to local ligand detachment from the interface or critical bond breaking. After 40 ns until simulation termination, the binding energy stabilized within a narrow range of −2 to 2 kcal/mol with significantly reduced fluctuations. The overall energy remained near zero, suggesting the complex ultimately adopted a weakly bound yet dynamically stable configuration.

RMSF analysis quantifies the conformational dynamics of protein residues. By comparing changes under different ligand-bound states, it reveals the regulatory mechanisms by which ligands modulate receptor dynamics, thereby linking these dynamics to biological functions and activities [43]. In the Octanal-OR6A2 and β-Selinene-OR6A2 complexes (Figure 6C), the RMSF values of residues fell within the relatively low ranges of 2.08 ± 1.38 Å and 2.46 ± 1.84 Å, respectively. This indicates minimal fluctuations in the protein backbone during simulations and overall conformation stability, suggesting the formation of stable binding patterns between ligands and receptors. Similarly, in the Octanal-OR6A2 and β-Selinene-OR6A2 complexes, RMSF values ranged from 2.7 Å to 26.0 Å and 3.6 Å to 18.2 Å, respectively. The protein core region (residues 20–100) in the Octanal-IGF1 complex exhibited generally lower RMSF values (mostly 2–11 Å), indicating structural rigidity. The N-terminal region (residues 1–20) exhibited higher RMSF (approximately 10–26 Å), reflecting its inherent structural flexibility. Fluctuations in the C-terminal region (residues beyond 120) showed a slight increase, consistent with the typically higher dynamic nature of terminal regions. Overall, the Octanal-IGF1 system exhibits RMSF values below 15 Å in key structural regions, indicating that Octanal binding does not induce global structural distortion or destabilization of IGF1. Instead, it may specifically anchor critical interface residues, inducing localized conformational rearrangements and enhancing structural compactness. The RMSF distribution for the β-Selinene-IGF1 system (3.6 Å to 18.2 Å) exhibits similar characteristics.

### 3.7. Limitations and Future Directions

Several considerations should be taken into account when interpreting the findings of this study. First, the ROAV approach assumes additive contributions of individual compounds. However, in real food systems, volatile compounds may interact through masking, synergy, or suppression effects, thereby influencing the actual perceived aroma. Furthermore, odor threshold values are typically determined in simple matrices (e.g., water or air) and may not fully reflect the complex matrix of ginseng products. Despite these limitations, ROAV analysis remains a widely accepted approach for identifying key aroma contributors in food products.

Second, computational prediction approaches provide a valuable foundation for identifying potential targets. Likewise, molecular docking and dynamics simulations offer important mechanistic insights into ligand–receptor interactions. Nevertheless, experimental validation using biological assays—such as in vitro binding assays or cellular functional studies—would further strengthen the biological relevance of these findings. Moreover, because these simulations are conducted under idealized conditions, they may not fully reflect the complexity of in vivo physiological environments.

Third, the relationship between aroma compound exposure and emotional responses was inferred from molecular interactions and sensory scores. Although these approaches provide useful preliminary insights, direct neurophysiological evidence—such as neurotransmitter release measurements or functional neuroimaging—would be valuable to further elucidate the underlying causal mechanisms.

Future research could focus on experimental validation of the predicted receptor–ligand interactions, exploration of additional olfactory and pleasure-related targets, and in-depth investigation of the neurophysiological mechanisms underlying aroma-induced emotional responses. Such efforts would further strengthen the translational potential of these findings for the development of evidence-based functional foods with mood-modulating properties.

## 4. Conclusions

This study established a comprehensive flavoromic and computational framework to decode the molecular basis of pleasant aroma perception in five processed ginseng products (FG, WG, DG, RG, and BG). Eight key pleasant aroma compounds—including hexanal, octanal, maltol, β-elemene, humulene, caryophyllene, β-selinene, and 5-methylfurfural—were identified as critical determinants of product differentiation. Multidimensional flavor analysis revealed a processing-driven aroma evolution from “green” to “roasted-medicinal” notes, with optimal pleasantness achieved at the RG stage, underscoring the importance of moderate processing in balancing flavor complexity and sensory acceptability. Mechanistically, computational simulations demonstrated that these compounds engage IGF1 and OR6A2 receptors through hydrogen bonds and hydrophobic interactions, thereby modulating olfactory and emotion-related pathways. Notably, aroma harmony, rather than intensity, emerged as the predominant driver of consumer preference, establishing a correlative bridge between molecular interactions and sensory quality. Molecular dynamics simulations further demonstrated the stable binding of β-selinene-IGF1 and octanal-OR6A2 complexes, elucidating the structural basis of ginseng’s pleasant aroma effects. Collectively, this work provides molecular-level evidence for the traditional Chinese medicine “nature and flavor” theory, suggests IGF1 as a potential key receptor for pleasantness, and offers a framework for developing evidence-based, mood-modulating ginseng products with tailored aroma profiles.

## Figures and Tables

**Table 1:** Odor descriptions, odor thresholds and ROAV of the main aroma actives in ginseng products.

ID	CAS	Threshold (μg/kg)	ROAV					Odor Description
			FG	WG	DG	RG	BG	
G2	66-25-1	4.5	3.83	10.07	9.03	6.98	3.85	fruity, woody, vegetative, leafy, grassy, sweaty, fresh
G5	98-00-0	2000	0.00	0.00	0.00	0.08	0.14	alcoholic, musty, sweet, caramel, bready, burnt
G7	111-27-3	500	0.04	0.00	0.00	0.00	0.00	green, sweet, savory, fruity, oily, woody
G9	111-71-7	0.006	302.50	1294.05	808.79	950.85	0.00	fresh, green, herbal, oily, grassy, fruity
G10	1192-62-7	1000	0.00	0.00	0.00	0.00	0.04	sweet, cocoa, caramel, coffee, nutty, almondy
G11	80-56-8	0.06	100.00	100.00	100.00	100.00	100.00	woody, sweet, fresh, resinous
G16	620-02-0	0.5	0.00	0.00	0.00	0.00	72.54	bready, sweet, caramel
G18	127-91-3	140	0.35	0.22	0.44	0.27	0.28	woody, minty, spicy, fresh, terpy
G19	124-13-0	0.58	48.51	45.95	29.88	40.28	0.00	waxy, citrus, herbal, fresh, green
G20	5989-27-5	34	0.11	0.11	0.13	0.20	0.41	citrus, fresh, sweet, terpy, lemon, tart
G23	122-78-1	4	0.00	0.00	0.00	0.12	0.00	green, sweet, honey, cocoa, chocolate, fruity, nutty
G27	1072-82-8	65	0.00	0.00	0.00	0.06	0.50	—
G28	111-87-5	125.8	0.02	0.00	0.00	0.00	0.00	green, sweet, waxy, fruity
G32	124-19-6	0.0003	6960.85	7813.84	19,637.36	17,692.21	27,514.62	fresh, waxy, green, lemon, citrus
G33	118-71-8	0.21	0.00	0.00	0.00	202.20	804.64	fruity, baked, bready, sweet
G36	18829-56-6	0.19	8.11	14.08	12.91	16.82	24.48	green, citrus, green, soapy, sweet
G37	112-44-7	0.00025	0.00	0.00	1055.64	1096.42	0.00	waxy, soapy, floral, citrus, green, fresh
G44	112-40-3	100	0.00	0.01	0.01	0.00	0.00	—
G45	112-31-2	3	1.73	0.73	0.97	0.89	0.99	waxy, green, sweet, floral, citrus, fresh
G51	3913-81-3	0.0003	0.00	570.03	0.00	0.00	1490.63	waxy, green, fruity, greasy, orange, citrus
G65	515-13-9	200	1.46	2.07	1.57	2.06	2.65	sweet
G73	6753-98-6	160	1.29	1.41	1.36	1.22	1.68	woody
G75	87-44-5	64	4.04	4.18	3.49	3.33	4.25	woody, spicy, nutty, oily, sweet, peppery
G77	17066-67-0	1	68.01	66.03	61.86	62.10	51.71	herbal
G88	58893-88-2	50	0.00	0.25	0.22	0.00	0.00	—

Odor description from flavornet database (http://www.flavornet.org, http://www.thegoodscentscompany.com). “—” indicates that the odor description for the compound was not found.

**Table 2:** The molecular docking results between receptors (OR6A2 and IGF1) and ligands.

Ligand	Receptor	Mean RMSD (Å)	Binding Energy (kcal/mol)	Hydrogen Bonds	Hydrophobic Interactions
Octanal	OR6A2	1.32	−3.74	LYS164	TYR74, PHE109, LEU110, PHE256, TYR283
	IGF1	1.29	−2.65	LEU112	TYR79, PRO111
β-Elemene	OR6A2	0.07	−5.20	—	GLU42, LEU45, ILE46, PRO292, ILE293, LEU297, LYS303, LEU306, LEU310
	IGF1	0.00	−4.52	—	LYS75, THR77, ALA86, GLN88
Humulene	OR6A2	0.00	−5.33	—	VAL77, LYS81, ILE97, THR104, GLN105
	IGF1	0.00	−5.08	—	PHE42, LEU53, ASP60, PHE64
Caryophyllene	OR6A2	0.00	−5.47	—	VAL77, PRO80, THR104, GLN105
	IGF1	0.00	−5.20	—	TYR79, GLU106, PRO111
β-Selinene	OR6A2	0.00	−6.93	—	TYR74, PHE109, LEU110, LEU212, PHE256, TYR283
	IGF1	0.00	−5.09	—	TYR79, GLU106, PRO111
Hexanal	OR6A2	0.97	−3.34	TYR74	ALA259, TYR283
	IGF1	0.88	−2.75	THR52	LEU53
5-Methylfurfural	OR6A2	0.10	−3.97	—	PHE256, TYR283, VAL287, PRO288
	IGF1	0.04	−3.47	GLU51	LEU53
Maltol	OR6A2	0.00	−4.20	VAL10, LEU171, TYR173	—
	IGF1	0.00	−3.97	ASN74, LYS75, THR89	LYS75

Hydrogen bonds and hydrophobic interactions are listed by residue name and number. “—“ indicates no interaction of that type was detected.

## Data Availability

The data presented in this study are available on request from the corresponding authors due to privacy concerns.

## Supplementary Materials

The following supporting information can be downloaded at https://www.mdpi.com/article/10.3390/foods15081337/s1. Table S1: Grid docking parameters in molecular docking; Table S2: Volatile components of ginseng products; Table S3: Olfactory and pleasantness attribute scores of five ginseng products; Table S4: Potential targets for receptors and ligands.
